# Medical Cannabis Use and Healthcare Utilization Among Patients with Chronic Pain: A Causal Inference Analysis Using TMLE

**DOI:** 10.3390/pharmacy13040096

**Published:** 2025-07-15

**Authors:** Mitchell L. Doucette, Emily Fisher, Junella Chin, Panagiota Kitsantas

**Affiliations:** 1 Health Economics and Outcomes Research Department, Leafwell, Miami, FL 33156, USA; 2 Department of Health Administration and Policy, George Mason University, Fairfax, VA 22030, USA

**Keywords:** medical marijuana, supervised machine learning, causality, chronic pain

## Abstract

Introduction: Chronic pain affects approximately 20% of U.S. adults, imposing significant burdens on individuals and healthcare systems. Medical cannabis has emerged as a potential therapy, yet its impact on healthcare utilization remains unclear. Methods: This retrospective cohort study analyzed administrative data from a telehealth platform providing medical cannabis certifications across 36 U.S. states. Patients were classified as cannabis-exposed if they had used cannabis in the past year, while unexposed patients had no prior cannabis use. Outcomes included self-reported urgent care visits, emergency department (ED) visits, hospitalizations, and quality of life (QoL), measured using the CDC’s Healthy Days measure. Targeted Maximum Likelihood Estimation with SuperLearner estimated causal effects, adjusting for numerous covariates. Results: Medical cannabis users exhibited significantly lower healthcare utilization. Specifically, exposure was associated with a 2.0 percentage point reduction in urgent care visits (95% CI: −0.036, −0.004), a 3.2 percentage point reduction in ED visits (95% CI: −0.051, −0.012) and fewer unhealthy days per month (−3.52 days, 95% CI: −4.28, −2.76). Hospitalization rates trended lower but were not statistically significant. Covariate balance and propensity score overlap indicated well-fitting models. Conclusions: Medical cannabis use was associated with reduced healthcare utilization and improved self-reported QoL among chronic pain patients.

## 1. Introduction

Estimates indicate that approximately 20% of adults in the United States experience chronic pain, translating to over 50 million individuals [1,2,3]. Chronic pain is especially burdensome among older adults, where it is often referred to as a “silent epidemic” because of its substantial link to poorer health, increased disability, and higher healthcare utilization [4]. Given these consistently high prevalence rates and the substantial individual and societal toll, chronic pain remains a critical public health issue in the U.S., warranting continued research aimed at refining epidemiologic estimates and optimizing treatment strategies.

Chronic pain exerts a profound toll on both individuals and health systems alike, generating substantial direct medical expenses and indirect societal burdens [5,6,7,8]. Chronic pain patients are more likely to use emergency and urgent care services, driven in part by unmanaged or worsening pain and complicated by comorbidities such as mood or metabolic disorders [8,9]. Moreover, older adults with chronic pain who rely on Medicare demonstrate similar patterns of elevated spending for hospitalizations and outpatient care [10,11].

Across numerous meta-analyses of non-pharmacological and pharmacological interventions for chronic pain, research suggests that while many approaches confer statistically significant benefits, clinically meaningful improvements are often modest or short-lived. For instance, acupuncture may outperform compared to no-treatment groups for low-back pain but shows only small or uncertain advantages over sham acupuncture [12] and therapeutic ultrasound yields minimal gains beyond placebo for chronic low-back pain [13]. Pharmacological treatments display efficacy, yet concerns about tolerability and only moderate effect sizes temper enthusiasm [14]. Similarly, medical devices such as transcutaneous electrical nerve stimulation or pulsed electromagnetic therapy may help some patients but lack robust long-term data, especially regarding functional outcomes [15]. Exercise-based therapies, mindfulness-based interventions, and acceptance and commitment therapy appear beneficial for quality of life (QoL) and psychological distress, albeit with smaller or less consistent effects on pain intensity itself [16,17,18]. Dry needling for fibromyalgia has likewise demonstrated short-term improvements in pain and associated symptoms, though the overall certainty of evidence remains moderate at best [19]. Finally, emerging digital solutions for self-management and telemedicine show promise for expanding access and offering structured behavioral support, but more research is needed to pinpoint long-term effectiveness and cost efficiency [20].

Despite these clinical benefits, data on whether such effects translate into meaningful reductions in downstream healthcare costs remain limited. Cost–utility studies of surgical options, for instance, show that lumbar interbody fusion can yield favorable cost per quality-adjusted life year (QALY), but outcomes vary widely based on the specific surgical approach and methodological assumptions [21]. Meanwhile, an observational cohort of adults who participated in an interdisciplinary program for chronic back pain saw reduced opioid prescriptions, fewer emergency department visits, and decreased imaging use at 12 months, suggesting a potential shift away from low-value services [22]. Taken together, these analyses underscore the need for expanded and higher-quality economic evaluations to understand how pain management strategies can balance clinical improvement with sustainable, value-based utilization of healthcare resources.

Access to and use of medical cannabis for the treatment of chronic pain has increased in the past decade in the United States. Currently, there are 36 states and the District of Columbia with medical cannabis programs, all of which include chronic pain as a qualifying medical condition [23,24]. Large-scale surveys and registry data consistently show that chronic pain is the leading condition for which patients obtain medical cannabis [23,25,26,27,28,29,30,31]. Although exact prevalence estimates vary by state and by program maturity, chronic pain regularly outpaces conditions like anxiety, cancer-related symptoms, and post-traumatic stress disorder in prompting new patient enrollments [31].

The impact of medical cannabis is most studied through two of its primary constituents, delta-9-tetrahydrocannabinol (THC) and cannabidiol (CBD). Medical cannabis exerts many of its analgesic effects through the CB1 receptor, a primary target for THC within the central nervous system [32]. By acting as a partial agonist at this receptor, THC can modulate both excitatory and inhibitory signaling, which leads to diminished nociceptive transmission [33]. In addition, such receptor engagement appears to reduce neuroinflammatory cascades by influencing immunomodulatory pathways [34]. Observational findings suggest that this mechanism often correlates with reductions in reported pain severity and better functional outcomes [35,36].

Although THC’s interaction with CB1 has been relatively well characterized, CBD appears to operate along a more indirect route. Several lines of investigation propose that CBD exerts minimal direct affinity for CB1 or CB2, but can modify endocannabinoid signaling by affecting the breakdown of endogenous ligands [32,33]. Some evidence also points to CBD acting as a negative allosteric modulator of CB1, which may help fine-tune the receptor’s overall contribution to pain relief [34,35].

Current evidence suggests medical cannabis is likely efficacious in addressing chronic pain. The 2017 National Academies of Science (NAS) report on the health impact of medical cannabis found that there was substantial or conclusive evidence of positive efficacy for chronic pain [37]. The findings of the NAS report confirmed what subsequent observational studies have underscored: many patients report moderate improvements in both pain intensity and related quality-of-life measures when cannabinoids are added to their treatment plans [38,39,40,41,42]. Specifically, a 2024 meta-analysis of randomized control trials found moderate to high certainty evidence of small to very small improvements in pain relief, physical functioning, and sleep quality among chronic pain patients [42]. The authors also noted several transient adverse side effects compared to placebo groups.

Research focused on the impact of medical cannabis on second-order outcomes has found possible substitution of medical cannabis for prescription analgesics, especially opioids, citing equivalent or improved pain relief coupled with fewer adverse effects [43,44,45,46,47,48,49,50,51,52]. Additionally, emerging research finds medical cannabis is likely a cost-effective treatment option in the treatment of noncancer chronic pain [53,54]. Yet gaps remain in the literature as most studies rely on small sample sizes and there is no link between medical cannabis use and downstream health economic implications, such as healthcare utilization rates.

Chronic pain affects an estimated 50 million US adults each year and contributes substantially to poor quality of life and healthcare costs. Despite growing clinical interest in medical cannabis as an analgesic alternative, few large observational studies have applied robust causal inference methods to quantify its real-world impact on quality of life and healthcare utilization. In this study, we apply Targeted Maximum Likelihood Estimation within a SuperLearner framework to estimate the effect of medical cannabis certification on quality of life and healthcare utilization among adults with chronic pain. We hypothesize that, compared with cannabis-naïve patients, certified medical cannabis patients who used for one year will experience significantly fewer urgent care, emergency department, and hospital visits as well as better quality of life. Thus, our objectives were the following:

(1) Estimate the Average Treatment Effect of medical cannabis use on patient-reported quality of life (QoL).
(2) Estimate the Average Treatment Effect of medical cannabis use on healthcare utilization, including urgent care, emergency department, and hospital visits.
(3) Estimate the Relative Risk of healthcare utilization outcomes among medical cannabis-exposed verses cannabis-naïve patients.

## 2. Materials and Methods

### 2.1. Study Design and Population

This retrospective cohort study utilized administrative data from Leafwell, a telehealth platform providing medical cannabis certifications across 36 U.S. states. The dataset included structured, self-reported health and demographic information collected at the time of patient certification or recertification of their medical cards. A previous study has highlighted the characteristics of this dataset in relation to medical cannabis patients with post-traumatic stress disorder [55], but the current study expands upon this work by incorporating a targeted analysis of healthcare utilization and QoL outcomes among chronic pain patients.

To establish a temporal component, we categorized patients into two exposure groups based on their reported cannabis use over the past year. The cannabis-exposed group included individuals who had used medical cannabis within the prior year and were seeking recertification of their medical card through Leafwell, while the unexposed group comprised first-time Leafwell patients who self-reported no cannabis use in the past year. By restricting the cannabis-exposed group to individuals returning for certification after at least one year of prior medical cannabis use, we strengthened the temporal aspect of exposure classification. Patients who had previously used cannabis recreationally or for medical reasons outside of their Leafwell medical card certification were excluded. The study used administrative data collected between 15 June and 15 September 2024.

For study inclusion, eligible patients were adults aged 18 years or older whose primary qualifying condition for obtaining a medical card was chronic noncancer pain. The sample included 5242 chronic pain patients, of whom 3943 had prior cannabis use and were seeking recertification (cannabis-exposed group), while 1299 had no prior cannabis exposure and were part of the unexposed group. Only 2.2% of patients had missing data and we therefore conducted a complete-case analysis.

### 2.2. Outcomes and Exposure Definition

Healthcare utilization was assessed through three self-reported binary outcomes, each reflecting whether the respondent had sought medical care in the past six months due to chronic pain-related symptoms. These outcomes were as follows: (1) at least one urgent care visit, (2) at least one emergency department (ED) visit, and (3) at least one hospital admission. All patients, regardless of cannabis use, were asked, “In the past six months, can you tell us about any medical care you received related to your condition?” Response options included “Yes” or “No” for each outcome: “I went to urgent care because of my condition,” “I went to the emergency room because of my condition,” and “I was admitted to the hospital because of my condition.”

Additionally, we examined self-reported QoL using the CDC’s Healthy Days measure (CDC HRQOL-4) [56,57], which asks respondents to quantify the number of days in the past month they experienced poor physical or mental health. We used this measure in two separate ways. First, it was analyzed as a continuous outcome, representing the total number of unhealthy days per month. Second, we also transformed it into a binary variable, where “0” indicated the patient had fewer than two unhealthy weeks (≤14 days) and “1” indicated the patient had more than two unhealthy weeks (>14 days) in the past month, for use in our healthcare utilization outcome models to aid model specification (i.e., propensity score matching).

We adjusted for a range of covariates known to influence both medical cannabis use and healthcare utilization. These included demographic variables (age, sex, race/ethnicity, and health insurance), lifestyle factors (smoking status and alcohol consumption), and health-related characteristics (chronic pain severity). For the binary healthcare utilization outcome models, we used the binary QoL measure noted above to control for overall health status. Age was categorized into quintiles to allow for non-linear effects (See Supplemental Figure S1 for the distribution of age by quintiles). Sex was classified as male or female, and race/ethnicity was dichotomized as White, non-Hispanic vs. all other racial/ethnic groups due to sample size considerations. Health insurance status was included in the outcome model but excluded from the treatment model, as health insurance status is independent of medical cannabis certification requirements. We accounted for smoking status and alcohol consumption given their associations with healthcare utilization. Smoking status was classified as current smoker vs. non-smoker. Alcohol use was categorized as current drinker vs. nondrinker. Chronic pain severity was assessed using the Graded Chronic Pain Scale-Revised (GCPS-R) and categorized as mild vs. bothersome/high-impact pain [58]. Because overall missingness was low (2.2%) (see Table 1), we applied a complete-case analysis.

### 2.3. Statistical Analysis

To estimate the causal effect of medical cannabis use on healthcare utilization and QoL, we employed Targeted Maximum Likelihood Estimation (TMLE) [59,60,61,62] with SuperLearner [63,64], a semi-parametric, doubly robust method designed to improve estimation efficiency while reducing reliance on strong parametric assumptions. TMLE combines machine learning algorithms with targeted adjustments to correct for potential bias, ensuring that the estimated treatment effects remain both flexible and efficient. Unlike traditional parametric models, TMLE does not require a correctly specified functional form for both the treatment and outcome models. Instead, it maintains consistency as long as either the treatment model or the outcome model is correctly specified, offering a key advantage in observational data where model misspecification can introduce bias [61].

We implemented the TMLE by first estimating the treatment assignment mechanism, known as the g-model, which predicted the probability of medical cannabis exposure based on baseline covariates. Simultaneously, the outcome model, or Q-model, was fitted to predict healthcare utilization and unhealthy days under both cannabis-exposed and unexposed conditions. To avoid model misspecification, we used the SuperLearner algorithm, or stacked ensemble machine learning, to estimate the initial models. By systematically assigning weights to multiple machine learning models through cross-validation, SuperLearner minimizes the risk of relying on a single, potentially misspecified model.

We conducted all TMLE and SuperLearner steps in R (v4.3.1 [65]) using the tmle [66] and SuperLearner [67] packages. After selecting Y (outcome), A (treatment), and W (baseline covariates) we dropped observations with any missing covariate values. Our SuperLearner library (sl_lib_expanded) comprised four learners, XGBoost, Random Forest, Generalized Additive Models, and Multivariate Adaptive Regression Splines [62], each applied to both the Q-model and the g-model with ten-fold cross-validation (cvQinit = TRUE) and verbose = TRUE to track fitting. After obtaining initial predictions, TMLE applied a targeted update to the outcome model, fine-tuning estimates to ensure efficiency and unbiasedness given the observed data. Following TMLE, we extracted the propensity scores (g1W) and computed inverse-probability weights (1/PS for treated, 1/(1 − PS) for controls). We then computed standardized mean differences before and after weighting and plotted the density of TMLE-derived propensity scores by treatment status to assess the positivity assumption.

The analysis focused on several key measures of treatment effect, including the Average Treatment Effect (ATE), the Average Treatment Effect among the Treated (ATT), and the Average Treatment Effect among the Unexposed (ATC). These estimates provided insight into the impact of medical cannabis use across different populations. For the binary outcomes, we also calculated Relative Risk (RR) to further contextualize the magnitude of the treatment effect. Variance estimates and 95% confidence intervals were derived using an influence function-based variance estimation approach, which accounts for the complexities of the TMLE algorithm and ensures accurate statistical inference. All baseline covariates were included in all TMLE models.

To assess model performance, we examined multiple diagnostic measures. First, we evaluated the SuperLearner model weights for both the treatment and outcome models to determine which algorithms contributed most heavily to the final estimations. We calculated standardized mean differences (SMDs) before and after TMLE weighting, with an SMD < 0.10 denoting acceptable covariate overlap, considering an SMD of less than 0.1 as indicative of strong covariate balance [68]. To assess the positivity assumption, we examined propensity score distributions in both the cannabis-exposed and cannabis-naïve groups.

All patient data were de-identified prior to analysis. The study received an exemption from ethical review by an independent Institutional Review Board (BRANY, IRB Number: IRB00000080). Patients consented to the aggregate use of their questionnaire data in accordance with Leafwell’s terms of service.

## 3. Results

Prior to analysis, there were notable differences between the cannabis-exposed and the unexposed group (Table 1). Specifically, among all covariates, only sex and current smoking status were not statistically significantly different (*p*-value > 0.05). All other covariates suggested statistically significant differences between cannabis-exposed and unexposed groups. Specifically, the cannabis-exposed group had a higher proportion of white, non-Hispanic (77.1% vs. 69.4%), was less likely to refrain from alcoholic drinking in the past 7 days (34.4% vs. 44.8%), was younger (age quintile 1: 17.9% vs. 28.3%), had a higher percentage of patients reporting two or less unhealthy weeks per month (47.9% vs. 23.8%), had a higher percentage of patients reporting less bothersome/high-impact chronic pain (mild chronic pain: 41.4% vs. 14.7%), and had higher health insurance coverage (87.6% vs. 84.6%). The unexposed group was generally more likely to have received urgent care, visited ED, and been hospitalized at least once in the past 6 months compared to the cannabis-exposed group (Table 1).

### 3.1. Average Treatment Effects of Medical Cannabis

Results of the TMLE with SuperLearner suggest medical cannabis exposure is associated with significant reductions in healthcare utilization (see Table 2). The ATE for urgent care visits was −0.020 (95% CI: −0.036, −0.004), indicating a 2.0 percentage point absolute reduction in probability among cannabis-exposed patients (a 26.8% reduction). The ATE for ED visits was slightly larger at −0.032 (95% CI: −0.051, −0.012), suggesting a 3.2 percentage point reduction in emergency visits attributable to cannabis use (a 32.9% reduction). Hospitalizations exhibited a non-significant ATE (−0.010, 95% CI: −0.023, 0.003), indicating medical cannabis use was not associated with changes in hospital admissions. For unhealthy days, medical cannabis use was associated with a significant improvement in self-reported health, with an ATE of −3.52 days per month (95% CI: −4.28, −2.76), an 18.1% reduction. We provide the average predicted probabilities of our outcomes among the cannabis-exposed and the unexposed groups in Supplemental Table S1. 

Treatment effects were also assessed using the ATT and ATC. The ATT estimates, which reflect the effect of cannabis exposure on those exposed, were consistent with ATE estimates: ATT = −0.017 for urgent care (95% CI: −0.033, −0.001), ATT = −0.034 for ED visits (95% CI: −0.055, −0.013), and ATT = −3.59 for unhealthy days (95% CI: −4.42, −2.77). Similarly, the ATC estimates, which estimate the potential effect of medical cannabis if applied to the unexposed population, showed comparable reductions (Table 2).

RR estimates further contextualized these findings (Table 2). The risk of urgent care visits was 27% lower for those exposed to medical cannabis compared to the unexposed (RR = 0.732, 95% CI: 0.577, 0.928), while the risk of ED visits was 33% lower (RR = 0.671, 95% CI: 0.533, 0.844). The relative reduction in hospitalizations was smaller (RR = 0.812, 95% CI: 0.621, 1.062) and did not reach statistical significance.

### 3.2. TMLE Model Performance and Covariate Balance

The SuperLearner ensemble models performed well in optimizing covariate balance and generating robust estimates of treatment effects (see Table 3). In the treatment model, Generalized Additive Models (GAMs) received the highest SuperLearner weight across all outcomes, particularly for ED visits (77.8%) and hospitalizations (86.3%). Extreme Gradient Boosting contributed meaningfully to predicting unhealthy days (17.8%), while Multivariate Adaptive Regression Splines (MARSs) played a minor role in several models.

For the Outcome models, GAMs also received the highest weight across all outcomes, with particularly strong contributions to predicting ED visits (93.1%) and urgent care visits (82.9%). Random forests contributed to hospital visits (10.5%) and unhealthy days (18.3%), suggesting that tree-based models provided some additional predictive value in these contexts.

Standardized mean differences (SMDs) before and after TMLE weighting demonstrated that the weighting approach substantially improved covariate balance (see Table 4). Before adjustment, several covariates exhibited large imbalances, particularly chronic pain (SMD = 0.626) and unhealthy days (SMD = 0.519). After TMLE weighting, all post-weighting SMDs were significantly reduced, indicating strong covariate balance across cannabis-exposed and unexposed groups. There were only two instances, across all four outcome models, where a covariate’s SMD was above 0.05; the SMD for age quintile 3 was 0.0532 in the ED visit model, and the SMD for sex was 0.1038 in the hospital visit model.

**Figure 1:**
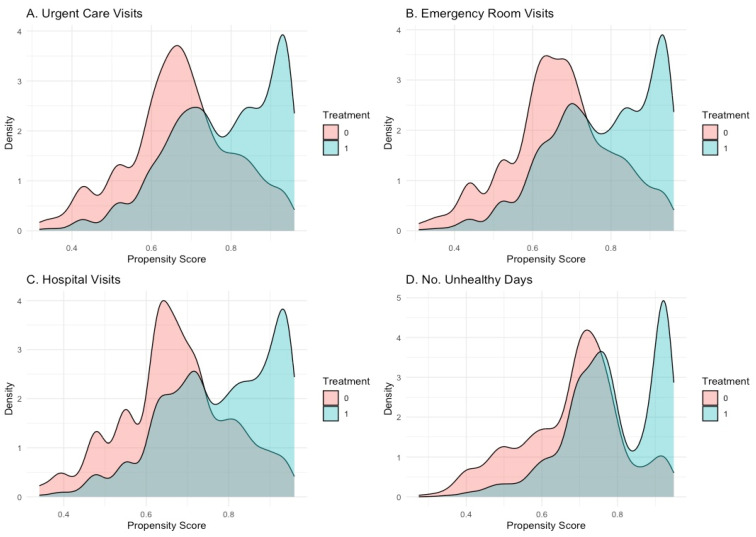
Propensity score overlap comparing cannabis-exposed and unexposed groups.

Propensity score distributions showed strong overlap between cannabis-exposed and unexposed groups across all four outcomes (See Figure 1). Density plots revealed that while some differences in distribution remained, particularly in the tails, there was sufficient overlap to support robust causal inference. This overlap, in conjunction with improved covariate balance, suggests that positivity assumptions were not violated, further strengthening confidence in the TMLE estimates.

## 4. Discussion

The ATE estimates indicated clinically meaningful decreases in multiple measures of healthcare utilization, including urgent care and ED visits, as well as fewer self-reported unhealthy days per month among the cannabis-exposed group. The associated RR estimates further substantiated these findings, revealing that cannabis-exposed patients had a 27% lower risk of requiring urgent care compared with non-cannabis-exposed individuals, while the relative reduction in ED visits approached one-third. Model diagnostics suggest strong covariate balance and propensity score overlap, underscoring the potential of medical cannabis to lessen short-term healthcare utilization in this patient population.

To our knowledge, this is the first study to provide effect estimates of medical cannabis exposure on healthcare utilization among chronic pain patients within a large population using real-world data. Additionally, our analysis provides a unique contribution to the literature in that it evaluates the effect of medical cannabis exposure on a self-reported health outcome and downstream healthcare utilization simultaneously. This allows us to better contextualize our findings. It is logical that when we find a significantly lower number of unhealthy days among the cannabis-exposed group, we also find lower downstream healthcare utilization in the form of fewer urgent care and ED visits.

These findings build on the established body of work demonstrating that medical cannabis can yield moderate but measurable improvements in QoL among chronic pain patients. Prior research found medical cannabis treatment was associated with patient-reported outcomes, specifically QoL [37,38,39,40,41,42]. Here, we find that among a large sample of chronic pain patients (*n* = 5242), those with a medical cannabis exposure for a year (*n* = 3943) had a significantly improved QoL using the CDC’s HRQOL-4 validated measure.

Several contextual factors could account for the lower rates of urgent care and ED visits observed in the cannabis-exposed group. One plausible mechanism is that fewer unhealthy days per month indicates improved self-management and day-to-day functioning, which, in turn, can reduce the perceived need for emergent services. Even modest improvements in pain or mental well-being could substantially lessen reliance on urgent healthcare resources when patients feel more confident in controlling their symptoms.

We asked patients to recall and self-report healthcare utilization from the past six months to determine usage rates. While this design may introduce recall bias (see Limitations below), it does provide a sufficiently long period for examining relevant associations. Prior interventions for chronic pain, such as acupuncture, therapeutic ultrasound [12,13], pharmacological therapies [14], and medical devices [15], have demonstrated some benefits but often result in only modest or short-lived improvements [12,13,14,15,16,17,18]. In this context, our findings add to the growing literature suggesting that medical cannabis not only improves QoL but may also reduce urgent healthcare use. Given the expanding body of evidence on its role in chronic pain treatment, further longitudinal studies are warranted to assess the long-term cost efficiency and real-world impact of medical cannabis.

Chronic pain affects roughly 20 percent of U.S. adults, over 50 million individuals, and is a leading driver of healthcare utilization [4]. Traditional therapies tend to yield modest or short-lived improvements in pain intensity and function, and many patients continue to rely on acute-care services despite multidisciplinary interventions [12,13,14,15,16,17,18,19,20]. In contrast, our TMLE analysis demonstrates that medical cannabis use is associated with 26.8% and 32.9% lower odds of having an urgent care or ED visit, alongside an 18.1% increase in patient-reported quality of life. These real-world gains build on prior work which finds small health improvements associated with medical cannabis use and noncancer chronic pain [42]. Taken together, these findings suggest that medical cannabis may offer a meaningful complement to existing pain management strategies by not only alleviating symptoms but also reducing the burden on acute-care systems.

Prior work has demonstrated that the adoption of medical cannabis laws is linked to lower health insurance premiums both in the individual market [69] and among commercial plans [24]. These savings have been attributed to reduced claims spending, but the specific pathways have remained unclear. Our analysis sheds light on one such mechanism: by certifying patients for medical cannabis, we observe meaningful reductions in urgent care and ED visits and improvements in patient-reported healthy days. Fewer acute-care encounters translate directly into lower aggregate claims for insurers. For self-insured employers, who bear the full cost of each utilization event, these decreases in utilization can help stabilize or reduce annual premium rates and stop-loss expenditures. In this way, expanded access to medical cannabis appears to offer a tangible lever for employers and insurers seeking to manage healthcare costs without compromising patient outcomes.

By leveraging a semi-parametric machine learning approach, TMLE allows for rigorous causal inference without imposing restrictive modeling assumptions. This is particularly important in studies of medical cannabis use, where individual health behaviors and clinical outcomes are influenced by a range of unobserved factors. The combination of machine learning, doubly robust estimation, and targeted updating provides a powerful framework for estimating treatment effects in observational data, ensuring that our findings reflect a well-calibrated balance between flexibility and statistical efficiency. Furthermore, our analysis uses data across a geographically diverse dataset [23], which increases our findings’ generalizability among chronic pain patients. But we must note that our reliance on data from a telemedicine platform may limit generalizability if individuals lacking internet access or those preferring in-person consultations differ in important ways from our study population.

Our analysis is not without limitations. The primary limitation this work faces is how we measured exposure temporality, as we assessed cannabis exposure differently between the cannabis-exposed and unexposed groups. The administrative data provides evidence of at least 1 years’ worth of medical cannabis exposure. However, we relied on self-reported data to determine exposure in our unexposed group. This may introduce social desirability bias, resulting in prior cannabis users identifying as cannabis-naïve and being improperly included in the analysis. This study is also hindered by its reliance on self-reported data specifically for our three healthcare outcomes. Patients may not have perfect recall of their prior 6-month healthcare utilization related to their chronic pain. Urgent care and ED visits are typically more acute events, potentially improving patient recall. Our use of the 30-day time span to quantify QoL through the CDC’s HRQOL-4 measure also introduces bias as it only represents a small portion of time as it relates to each patient’s health relative to the 6-month healthcare utilization questions. While our study does have strong data completion (missing data = 2.2%), our analysis did not account for differences in medical cannabis dosage, frequency, or route of administration. Future research ought to answer whether the effects found here are homogeneous across medical cannabis products and use habits.

## 5. Conclusions

The findings of this study suggest, in line with existing research, that medical cannabis is likely an effective treatment option for patients with chronic pain. Moreover, we found that, in addition to an increase in QoL, medical cannabis exposure is associated with lower risk of urgent care and ED visits, when comparing patients who used medical cannabis for at least one year to cannabis-naïve patients. This underscores the potential for not only QoL gains associated with medical cannabis use, but also positive downstream effects on the healthcare system resulting from treatment.

## Figures and Tables

**Table 1:** Descriptive statistics of cannabis-exposed and unexposed.

			Cannabis-Exposed *	Unexposed *	Total	Test Statistic	Missing
N (%)			3943 (75.2%)	1299 (24.8%)	5242		
Demographics							
	Sex					0.597	25
		Female	1693 (43.1%)	547 (42.3%)	2240 (42.9%)		
		Male	2231 (56.9%)	746 (57.7%)	2977 (57.1%)		
	Race/ethnicity					<0.001	
		All other race/ethnicities	903 (22.9%)	398 (30.6%)	1301 (24.8%)		
		White non-Hispanic	3040 (77.1%)	901 (69.4%)	3941 (75.2%)		
	Current Smoking Status					0.121	---
		No	3149 (79.9%)	1063 (81.8%)	4212 (80.4%)		
		Yes	794 (20.1%)	236 (18.2%)	1030 (19.6%)		
	No Alcoholic Drinks in the past 7 days?					<0.001	---
		No (Current drinker)	2587 (65.6%)	717 (55.2%)	3304 (63.0%)		
		Yes (Nondrinker)	1356 (34.4%)	582 (44.8%)	1938 (37.0%)		
	Age, Quintiles					<0.001	---
		Quintile 1	705 (17.9%)	367 (28.3%)	1072 (20.5%)		
		Quintile 2	873 (22.1%)	235 (18.1%)	1108 (21.1%)		
		Quintile 3	816 (20.7%)	236 (18.2%)	1052 (20.1%)		
		Quintile 4	775 (19.7%)	210 (16.2%)	985 (18.8%)		
		Quintile 5	774 (19.6%)	251 (19.3%)	1025 (19.6%)		
Health Status							
	Quality of Life, in number of unhealthy weeks					<0.001	---
		Two or less unhealthy weeks per month	1890 (47.9%)	309 (23.8%)	2199 (41.9%)		
		Three or more weeks unhealthy weeks per month	2053 (52.1%)	990 (76.2%)	3043 (58.1%)		
	Chronic Pain Severity					<0.001	91
		Mild Chronic Pain	1606 (41.4%)	187 (14.7%)	1793 (34.8%)		
		Bothersome or High-impact Chronic Pain	2270 (58.6%)	1088 (85.3%)	3358 (65.2%)		
	Health Insurance?					0.006	---
		No	490 (12.4%)	200 (15.4%)	690 (13.2%)		
		Yes	3453 (87.6%)	1099 (84.6%)	4552 (86.8%)		
Healthcare Utilization							
	Received urgent care at least one time in the past 6 months					<0.001	---
		No	3751 (95.1%)	1169 (90.0%)	4920 (93.9%)		
		Yes	192 (4.9%)	130 (10.0%)	322 (6.1%)		
	Received emergency room care at least one time in the past 6 months					<0.001	---
		No	3719 (94.3%)	1153 (88.8%)	4872 (92.9%)		
		Yes	224 (5.7%)	146 (11.2%)	370 (7.1%)		
	Was hospitalized at least one time in the past 6 months					<0.001	---
		No	3791 (96.1%)	1213 (93.4%)	5004 (95.5%)		
		Yes	152 (3.9%)	86 (6.6%)	238 (4.5%)		

Note: * Patients were classified as cannabis-exposed if they were medical cannabis users in the past year and were seeking recertification, while unexposed were first-time patients with no prior cannabis use. There were 25 missing data points for sex and 91 for the chronic pain severity variables, resulting in a total of 116 missing observations (2.2% of the sample). Test statistic was χ2 test.

**Table 2:** Impact of medical cannabis on healthcare utilization and quality of life, analyzed using Targeted Maximum Likelihood Estimation.

Outcome	ATE *			ATT *			ATC *			RR *		
	Est.	95% CI		Est.	95% CI		Est.	95% CI		Est.	95% CI	
Urgent Care	−0.020	−0.036	−0.004	−0.017	−0.033	−0.001	−0.029	−0.049	−0.009	0.732	0.577	0.928
ED Visits	−0.032	−0.051	−0.012	−0.034	−0.055	−0.013	−0.026	−0.048	−0.004	0.671	0.533	0.844
Hospital Visits	−0.010	−0.023	0.003	−0.008	−0.021	0.004	−0.015	−0.032	0.002	0.812	0.621	1.062
Unhealthy Days	−3.517	−4.280	−2.755	−3.591	−4.416	−2.766	−3.311	−3.970	−2.652	NA	NA	NA

Note: * Average Treatment Effect (ATE), Average Treatment Effect among the Treated (ATT), Average Treatment Effect among the Unexposed (ATC), and Relative Risk (RR). RR not provided for the continuous outcome Unhealthy Days.

**Table 3:** Treatment and outcome model weights from the TMLE SuperLearner algorithm.

Treatment model SuperLearner weights				
	Urgent Care	ED Visits	Hospital Visits	Unhealthy Days
Extreme Gradient Boosting	0.0413	0.1538	0.1371	0.1777
Random Forest	0.000	0.000	0.000	0.0304
Generalized Additive Models	0.7472	0.7784	0.8629	0.7655
Multivariate Adaptive Regression Splines	0.2115	0.0678	0.000	0.0265
Outcome model SuperLearner weights				
	Urgent Care	ED Visits	Hospital Visits	Unhealthy Days
Extreme Gradient Boosting	0.111	0.0177	0.000	0.0153
Random Forest	0.000	0.000	0.1049	0.1831
Generalized Additive Models	0.8294	0.9311	0.664	0.612
Multivariate Adaptive Regression Splines	0.0596	0.0512	0.231	0.1896

**Table 4:** Standardized mean difference (SMD) before and after TMLE weighting.

		Post-TMLE Standardized Mean Difference (SMD)			
		Outcome Models			
Covariates	Baseline SMD	Urgent Care	ED Visits	Hospital Visits	Unhealthy Days
Sex	0.015	0.0076	0.0004	0.1038	0.0037
Race/ethnicity	0.178	0.01	0.0081	0.0066	0.0019
Current smoking status	0.053	0.0097	0.0053	0.0064	0.0053
Alcohol consumption status	0.211	0.0312	0.0341	0.0378	0.0378
Health Insurance status	0.081	0.0244	0.0108	0.005	0.0116
Quality of life, in number of unhealthy weeks	0.519	0.0163	0.0155	0.0195	NA
Chronic pain status	0.626	0.0199	0.0204	0.0192	0.03
Age, Quintile 1	0.25	0.0076	0.0002	0.0033	0.0029
Age Quintile 2	0.103	0.0376	0.0304	0.0407	0.0312
Age Quintile 3	0.06	0.0336	0.0532	0.0257	0.0479
Age Quintile 4	0.094	0.0106	0.0308	0.0022	0.0285
Age Quintile 5	0.009	0.0071	0.0081	0.0139	0.0147

## Data Availability

The dataset presented in this article is not readily available because the data are proprietary and confidential property of Leafwell. Requests to access the dataset should be directed to the corresponding author.

## Supplementary Materials

The following supporting information can be downloaded at: https://www.mdpi.com/article/10.3390/pharmacy13040096/s1, Supplemental Table S1: Average predictive probability of exposure among the cannabis-exposed and unexposed groups; Supplemental Figure S1: Age distribution by quintiles.
