# Supplementary material for: Medical Cannabis Use and Healthcare Utilization Among Patients with Chronic Pain: A Causal Inference Analysis Using TMLE
Source: Pharmacy (Basel). 2025 Jul 15;13(4):96. doi: 10.3390/pharmacy13040096 (PMC12286269; doi:10.3390/pharmacy13040096)

Supplemental Table S1: Average predictive probability of exposure among the cannabis-exposed and unexposed groups

	**Cannabis-exposed***			**Unexposed**		
	Average Predictive Probability	95% Confidence Interval (CI)		*Average Predictive Probability*	95% CI	
**Outcome**		*Lower*	*Upper*		*Lower*	*Upper*
Urgent Care	0.0546	0.0469	0.0622	0.0746	0.0601	0.089
ED Visits	0.0642	0.0558	0.0726	0.0957	0.0775	0.1138
Hospital Visits	0.0424	0.0356	0.0491	0.0522	0.0408	0.0636
Unhealthy Days	15.861	15.515	16.208	19.378	18.676	20.081

Note: *Patients were classified as cannabis-exposed if they were medical cannabis users in the past year and were seeking recertification, while unexposed were first-time patients with no prior cannabis use.

Supplemental Figure S1: Age distribution by quintiles

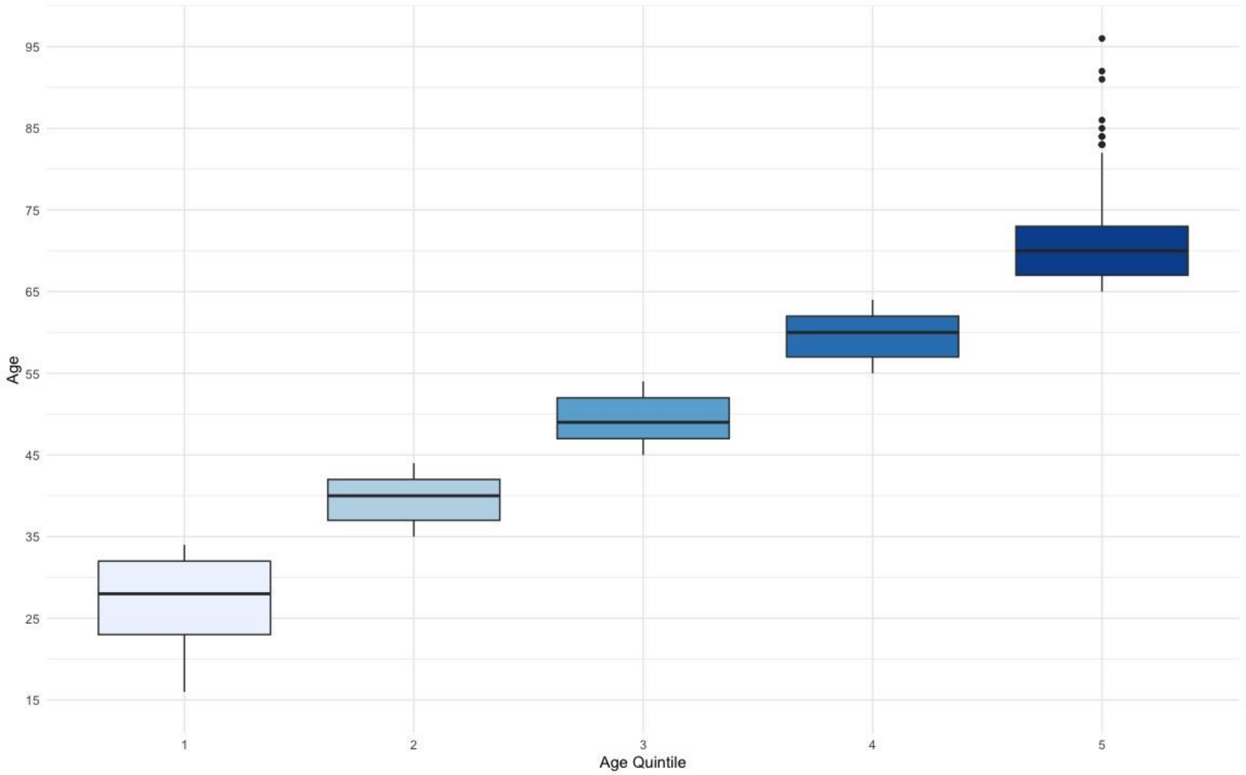

Supplement: Supplementary file 1 [file pharmacy-13-00096-s001.zip › pharmacy-3689214-supplementary.pdf]
